# Integrated bioinformatic analysis identifies GADD45B as an immune-related prognostic biomarker in skin cutaneous melanoma

**DOI:** 10.1186/s41065-025-00437-0

**Published:** 2025-05-11

**Authors:** Qing Zhang, Song He, Zhonghao Ji, Xiwen Zhang, Bao Yuan, Ruirui Liu, Yimin Yang, Yu Ding

**Affiliations:** 1 Department of Laboratory Animals, College of Animal Sciences, Jilin University, Changchun, Jilin 130062 P.R. China; 2 Department of Thoracic Surgery, Institute of Thoracic Oncology, Frontiers Science Center for Disease-Molecular Network, West China Hospital of Sichuan University, Chengdu, 610097 P.R. China; 3 Department of Basic Medicine, Changzhi Medical College, Changzhi, Shanxi 046000 P.R. China; 4 Department of Intensive Care Unit, First Hospital of Jilin University, Changchun, Jilin 130021 P.R. China

**Keywords:** Drug resistance, GADD45B, Immune infiltration, Mechanism, Skin cutaneous melanoma (SKCM)

## Abstract

Skin cutaneous melanoma (SKCM) arises from melanocytes and is an aggressive form of skin cancer. If left untreated, most melanomas will metastasize, posing a major health risk. GADD45B, a member of the GADD45 family, is known to be involved in DNA damage repair; however, its specific role in SKCM remains largely unclear. In this study, we comprehensively investigated the function of GADD45B in SKCM. By integrating 26 SKCM-related datasets from The Cancer Genome Atlas (TCGA), Cancer Cell Line Encyclopedia (CCLE), cBioPortal for Cancer Genomics (cBioPortal), Gene Expression Omnibus (GEO), and other databases, we conducted functional enrichment, immune infiltration, and single-cell analyses using R. Additionally, transcriptome sequencing of 30 human SKCM cell lines, phenotype characterization of 29 SKCM lines in vitro, and macrophage polarization analysis were performed. We found that GADD45B expression was significantly downregulated in SKCM patients compared to normal controls (*p* < 0.001), and higher GADD45B levels correlated with better prognosis (*p* < 0.05). GADD45B also showed high diagnostic accuracy, with an area under the curve (AUC) of 0.986. GO and KEGG analyses revealed a strong association between GADD45B and immune-related pathways. Gene Set Variation Analysis (GSVA) and single-cell sequencing suggested that GADD45B may serve as a novel immune checkpoint, predominantly expressed in macrophages and promoting M1 polarization. In vitro, overexpression of GADD45B significantly inhibited SKCM cell proliferation, potentially via suppression of the PI3K/Akt signaling pathway, and also reduced chemotherapy resistance. Furthermore, in vivo experiments using a xenograft mouse model demonstrated that GADD45B overexpression significantly suppressed tumor growth. Mice injected with GADD45B-overexpressing tumor cells exhibited smaller tumor volumes from day 15 onwards compared to controls, with markedly reduced tumor volume and weight at the endpoint. These results underscore the potential of GADD45B as an effective tumor suppressor in SKCM. In conclusion, our findings highlight GADD45B as a key regulator in SKCM progression, capable of restraining tumor cell proliferation and enhancing apoptosis. GADD45B holds promise as a novel diagnostic and prognostic biomarker and a potential target for SKCM immunotherapy.

**Supplementary Information:**

The online version contains supplementary material available at 10.1186/s41065-025-00437-0.

## Introduction

Melanoma develops from the malignant transformation of melanin-producing melanocytes and is called skin cutaneous melanoma (SKCM) if it is located in the basal layer of the skin epidermis [1]. SKCM is one of the most aggressive skin cancers and one of the major causes of death due to its metastasis [2]. Moreover, the incidence of SKCM is increasing globally, with Australia/New Zealand having the highest incidence, with 42/100,000 males and 31/100,000 females having SKCM [3]. Advanced melanoma is very aggressive and insensitive to chemotherapy and radiation, and surgical excision is often the preferred treatment for patients with primary melanoma [4]. Studies have shown that a high level of immune cell infiltration is associated with a good prognosis in patients [5]. Molecular therapy targeting immune checkpoints (such as anti-PD1/PDL1, anti-CTLA-4, and anti-TIM3) has a certain effect on improving the condition of patients with metastatic melanoma [6, 7], but there are still some patients in which the therapeutic effects are poor [8]. Consequently, the identification of diagnostic and prognostic biomarkers with superior specificity and sensitivity is imperative for the optimization of therapeutic interventions in SKCM.

The growth arrest DNA damage-inducible gene 45 (GADD45) family includes the GADD45A, GADD45B and GADD45G genes, which are involved in DNA repair, cell survival, senescence and apoptosis [9, 10]. The proteins encoded by the GADD45 gene family are highly conserved among different species [11, 12]. Studies have reported that GADD45B plays a therapeutic role in liver cancer [13], colorectal cancer [14], pituitary gonadotropin tumors [15] and nervous system diseases [16]. Emerging evidence suggests that GADD45B plays a significant role in modulating immune responses within the tumor microenvironment. Specifically, it has been shown to influence macrophage polarization towards the M1 phenotype, which is associated with anti-tumor immunity Connection to Immune-Oncology [9, 17]. In the context of immune-oncology, GADD45B’s modulation of immune cells, particularly macrophages, could provide critical insights into the development of new immune checkpoint inhibitors. Studies have highlighted the role of immune cell infiltration, particularly macrophages, in the prognosis of melanoma [10, 18–20]. Furthermore, GADD45B has demonstrated potential in overcoming drug resistance, a common challenge in melanoma treatment, by sensitizing tumor cells to chemotherapy [21], This dual role—both in immion and in combating drug resistance—positions GADD45B as a promising therapeutic target in SKCM.” Therefore, GADD45B might be essential for the survival of cancer cells. Nevertheless, more research is needed to determine GADD45B in SKCM patients.

The representation of GADD45B in SKCM and its association with clinical characteristics were examined in this study using data on GADD45B gene expression from the TCGA (the cancer genome atlas) and GEO (gene expression omnibus) databases as well as the associated clinical data. The R programming language was utilized for this analysis. The CCLE (cancer cell line encyclopedia) and cBioPortal databases were also used for auxiliary validation. The CIBERSORT algorithm and Seurat, Magrittr and Patchwork data packets were used to evaluate the correlation between the GADD45B gene and immune infiltration and to further verify its enrichment in macrophages and promotion of macrophage M1 polarization. ROC (receiver-operating characteristic) and Kaplan‒Meier survival analyses were used to clarify the diagnostic and prognostic value of GADD45B in SKCM patients. The interaction of GADD45B with related genes was further examined in in vitro tests and transcriptome sequencing to uncover a potential mechanism of action and to establish a theoretical framework for the therapeutic management of SKCM.

## Materials and methods

### Cell culture and data mining

The human malignant melanoma cell lines A375 and SK-MEL-1 were obtained from Boster (Wuhan, China) and Zhong Qiao Xin Zhou (Shanghai, China), respectively. A375 cells were cultured in DMEM (Sigma, USA) with 10% FBS (Lonsera, UY). SK-MEL-1 cells were cultured in MEM (MeilunBio, China) with 10% FBS (Lonsera, UY). The human myeloid leukemia monocyte cell line THP-1 was obtained from Boster (Wuhan, China) and cultured in RPMI 1640 medium (MeilunBio, China) with 10% FBS (Lonsera, UY) and 0.05 mM 2-mercaptoethanol (Aladdin, USA). Cells were differentiated into M0 macrophages by treatment with 100 ng/ml PMA (MCE, USA) for 48 h. Measurement of CD86 and CD206 expression by flow cytometry analysis to assess cell differentiation. Additionally, all cells were kept at 37 °C in a humidified incubator with 5% CO_2_.

The transcriptomic profiles and clinicopathological metadata for SKCM patients were sourced from repositories such as TCGA, GEO, cBioPortal, and CCLE. Histological evaluations of both normal and neoplastic tissues were procured from the Human Protein Atlas (HPA). Information pertaining to pharmacological responsiveness can be found in the Genomics of Drug Sensitivity in Cancer (GDSC) database.

### Creation and transfection of vectors

The GADD45B overexpression vector was constructed by amplifying the gene and cloning it into the NheI and HindIII sites of the pcDNA3.1(+) vector, yielding pcDNA3.1-GADD45B. Cells were transfected with Lipofectamine 2000 (Invitrogen, USA) by following the manufacturer’s protocol.

### RNA extraction and RT‒qPCR

RNA was isolated using TRNzol reagent (Life, USA) per the manufacturer’s instructions. cDNA was synthesized from 2 µg RNA per sample using the FastKing cDNA kit (TIANGEN, China). RT‒qPCR was performed on an Eppendorf qPCR system using a SYBR kit (TIANGEN, China).

### Cell viability assays

At 24 h posttransfection, 5 × 10^3^ cells per well were seeded in 96-well plates. At 0, 24, 48, 72 and 96 h, CCK8 reagent (10 µL; MCE) was added and incubated for 1 h before measuring OD450 on a microplate reader (TECAN, AUT).

### Wound healing assays

Confluent transfection-completed monolayers in 6-well plates were scratched with pipette tips, rinsed with PBS, and incubated in serum-free media. Imaging of the wound site at different time points was analyzed using ImageJ.

### Transwell assays

Cells were trypsinized into single cells (5 × 10^5^ cells/mL), and suspensions were made in serum-free medium. Then, 100 µL of cell suspension was added to the upper chamber of the Transwell, and 500 µL DMEM containing 10% FBS was added to the lower chamber. After 24 h, the upper chambers were washed with PBS, the cells were removed, and the remaining cells were fixed with 4% paraformaldehyde and stained with 0.1% crystal violet. Membranes were placed on slides, and migrated cells were imaged by inverted microscopy. The protocol for the invasion assay was essentially the same as that for the migration assay, except that Matrigel (ABW, China) was added to the upper chamber in advance.

### Cell cycle and apoptosis assays

The treated cells were processed according to the protocol using a cell cycle analysis kit (Beyotime, China) and apoptosis analysis kit (Absin, China), and then data were analyzed with flow cytometry (BECKMAN COULTER, USA) and Modifit software.

### Flow cytometry surface staining

According to the manufacturer’s protocol, the treated cell suspension was washed using PBS, resuspended, treated with membrane-breaking agent (#R30489), labeled with PE-conjugated anti-CD86 (#FHP086-01) and FITC-conjugated anti-CD206 antibodies (#FHF206-01), and later detected by flow cytometry. The antibody was purchased from 4 A Biotech., Ltd.

### Western blotting

Proteins were extracted with RIPA buffer (Beyotime, China) and quantified by BCA assay (Beyotime, China). Samples were separated by 15% SDS‒PAGE, transferred to PVDF (Immobilon-P, Ireland), and blocked with protein-free rapid blocking buffer (Epizyme, China) for 30 min at room temperature. Membranes were probed overnight (4 °C) with primary antibodies against GADD45B (1:1000, Baijia), p-PI3K, PI3K, p-Akt, Akt and GAPDH (1:1000, Affinity). Membranes were washed with TBST (Biosharp, China) and incubated (1 h, room temperature) with HRP-conjugated secondary antibodies (1:5000, Affinity). After a subsequent TBST rinse, protein bands were visualized using enhanced chemiluminescence (ECL) substrate (Beyotime, China) and captured on a fully automated chemiluminescence imaging system.

### Illumina sequencing

At 24 h posttransfection, RNA was extracted with TRIzol reagent (Life, USA) and quality-checked. Reverse transcription using the Illumina RNA-Seq Kit protocol generated cDNA libraries. Single-end 50 nt sequencing was performed on the Illumina NovaSeq 6000 (LC Bio, China). Sequencing data were generated as FPKM (fragments per kilobase exon per million reads) for each transcript and uploaded to the GEO dataset (ID: GSE221101) [22].

### GO/KEGG analysis

In this study, RNA-sequencing expression profiles and related clinical data of SKCM patients were downloaded from the TCGA database. Patients were categorized into high (*n* = 236) and low (*n* = 235) GADD45B expression groups based on median expression levels. Differentially expressed mRNAs were identified using the limma package, with an adjusted p-value < 0.05 (Benjamini-Hochberg correction) and|log2(FC)| > 1 as significance thresholds. Gene Ontology (GO) functional and Kyoto Encyclopedia of Genes and Genomes (KEGG) pathway enrichment analyses were conducted using the ClusterProfiler package in R, with an adjusted p-value < 0.05 as the threshold for significant enrichment. Background gene sets for enrichment analysis were derived from the full human genome as annotated in the org.Hs.eg.db database. The same analytical workflow was applied to our own sequencing dataset (GSE221101) to validate the findings.

### Gene set variation analysis (GSVA)

We identified immune-related genes from the Gene Set Enrichment Analysis (GSEA) website. Using default parameters in R, we computed the functional enrichment score for each SKCM sample to evaluate immune responses. The enrichment results were visualized as a heatmap using the pheatmap package in R. Pearson correlation analysis was performed to assess the association between GADD45B expression and immune responses in SKCM samples.

### Immunization and correlation analysis

RNA-sequencing expression data and related clinical information for SKCM patients were obtained from the TCGA dataset. To evaluate the reliability of the immune score assessment, we utilized the R package immuneeconv, which integrates six recent algorithms, including TIMER, xCell, MCP-counter, CIBERSORT, EPIC, and quanTIseq. Correlation between GADD45B expression and immune cell infiltration levels was analyzed using the Spearman correlation coefficient, with p-values adjusted using the Benjamini-Hochberg method to control the false discovery rate (FDR). Immune checkpoint expression levels were compared using Wilcoxon rank-sum tests, and results were visualized using ggplot2 and pheatmap packages in R. p-value < 0.05 was considered statistically significant.

### Analysis of single-cell clusters

Single-cell RNA-seq data from GSE72056 were obtained from GEO and analyzed using Seurat. Genes expressed in > 3 cells and ≥ 200 genes/cell were retained. Highly variable genes were identified using FindVariableFeatures and used for PCA. Clustering with FindClusters (resolution = 0.3) revealed nearest neighbors based on the first 20 PCA components. UMAP represents cell states in 2D. Cell types were assigned to clusters based on signature genes from CellMarker and the literature.

### Analysis of cell stemness

RNA-sequencing expression data and associated clinical information for SKCM patients were obtained from TCGA. OCLR calculated mRNA signature scores (mRNAsi) based on an 11,774-gene expression profile. Spearman correlation was used to analyze RNA expression data. The results were normalized by subtracting the minimum and then dividing by the maximum to map the dryness index to [0,1].

### Correlation analysis of IC50

Based on relevant data from SKCM patients obtained from TCGA, chemotherapy response prediction was performed using pRRophetic with GDSC data. The IC50 values were estimated by ridge regression with default parameters.

### Statistical analyses

Analyses were conducted in R. Kaplan‒Meier survival analysis was performed using the R package survival, and statistical significance was assessed using the log-rank test. Cox proportional hazards regression analysis was conducted to estimate hazard ratios (HR) and 95% confidence intervals (CI). The proportional hazards assumption was tested using Schoenfeld residuals.Spearman correlation was used to analyze nonnormally distributed variables. Student’s t tests and one-way ANOVA assessed differences between two and multiple groups, respectively. P values < 0.05 were considered significant.

## Results

### GADD45B is downregulated and has diagnostic and prognostic value for SKCM

**Fig. 1:**
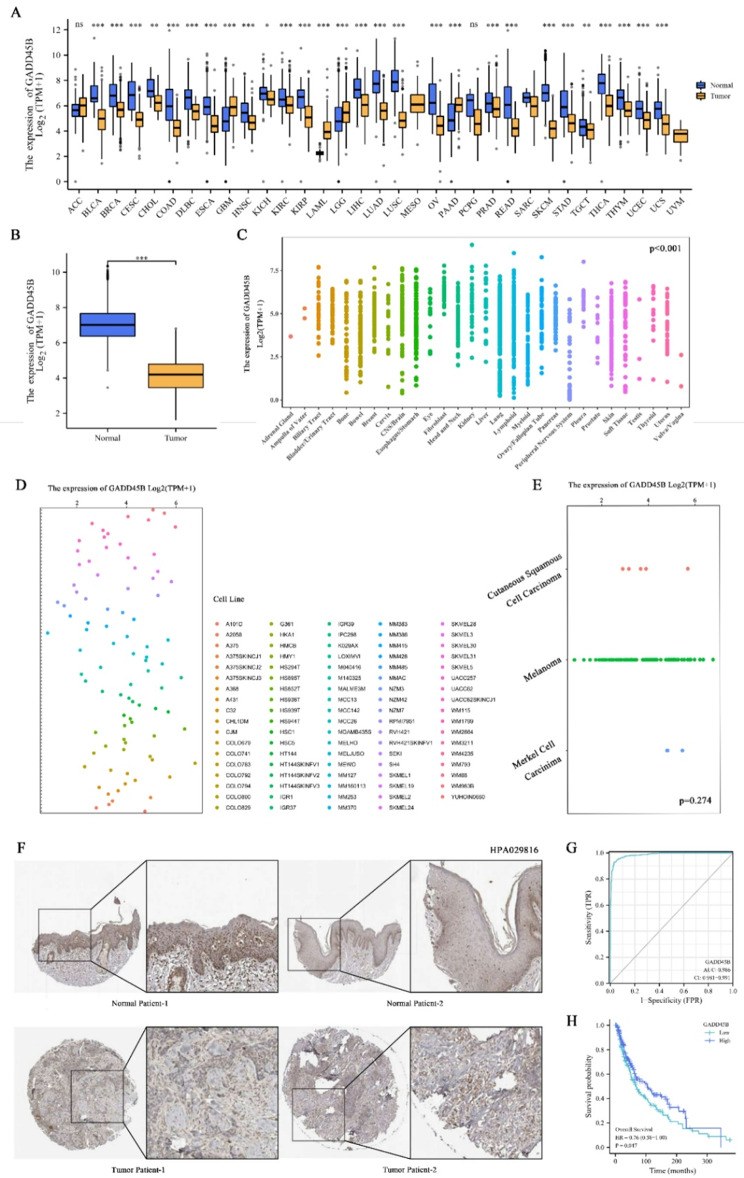
GADD45B is downregulated and has diagnostic and prognostic value for SKCM. (**A**) GADD45B expression in TCGA paracancers and tumors. (**B**) Low GADD45B levels in SKCM patients in the TCGA database. (**C**) GADD45B expression by lineage in the CCLE database. (**D**) GADD45B expression by skin cell line in the CCLE database. (**E**) GADD45B expression by primary disease in the CCLE database. (**F**) Lower GADD45B in SKCM than in normal patients according to the HPA database (HPA029816). (**G**) GADD45B as a diagnostic marker by ROC analysis (AUC, area under the curve). (**H**) High GADD45B correlated with better prognosis by Kaplan‒Meier curves in SKCM patients

TCGA was used to analyze GADD45B expression in normal and tumor tissues. The results showed that GADD45B expression was statistically significant among 28 groups, including BLCA and CHOL (*p* < 0.01) (Fig. 1A), and lower in SKCM compared to normal (*p* < 0.001) (Fig. 1B), which was confirmed by the clinicopathological sections in HPA (Fig. 1F). The analysis was extended using the CCLE database, which revealed significant differential expression in various tissues (Fig. 1C) but not in skin-associated cell lines (Fig. 1D, E). In addition, this study also explored the diagnostic and prognostic value of GADD45B in SKCM by using ROC (AUC = 0.986) and Kaplan‒Meier curves, respectively (Fig. 1G, H), and its higher expression was associated with a higher survival rate in SKCM patients (*p* < 0.05).

### Analysis of GADD45B and clinical factors in SKCM patients

**Fig. 2:**
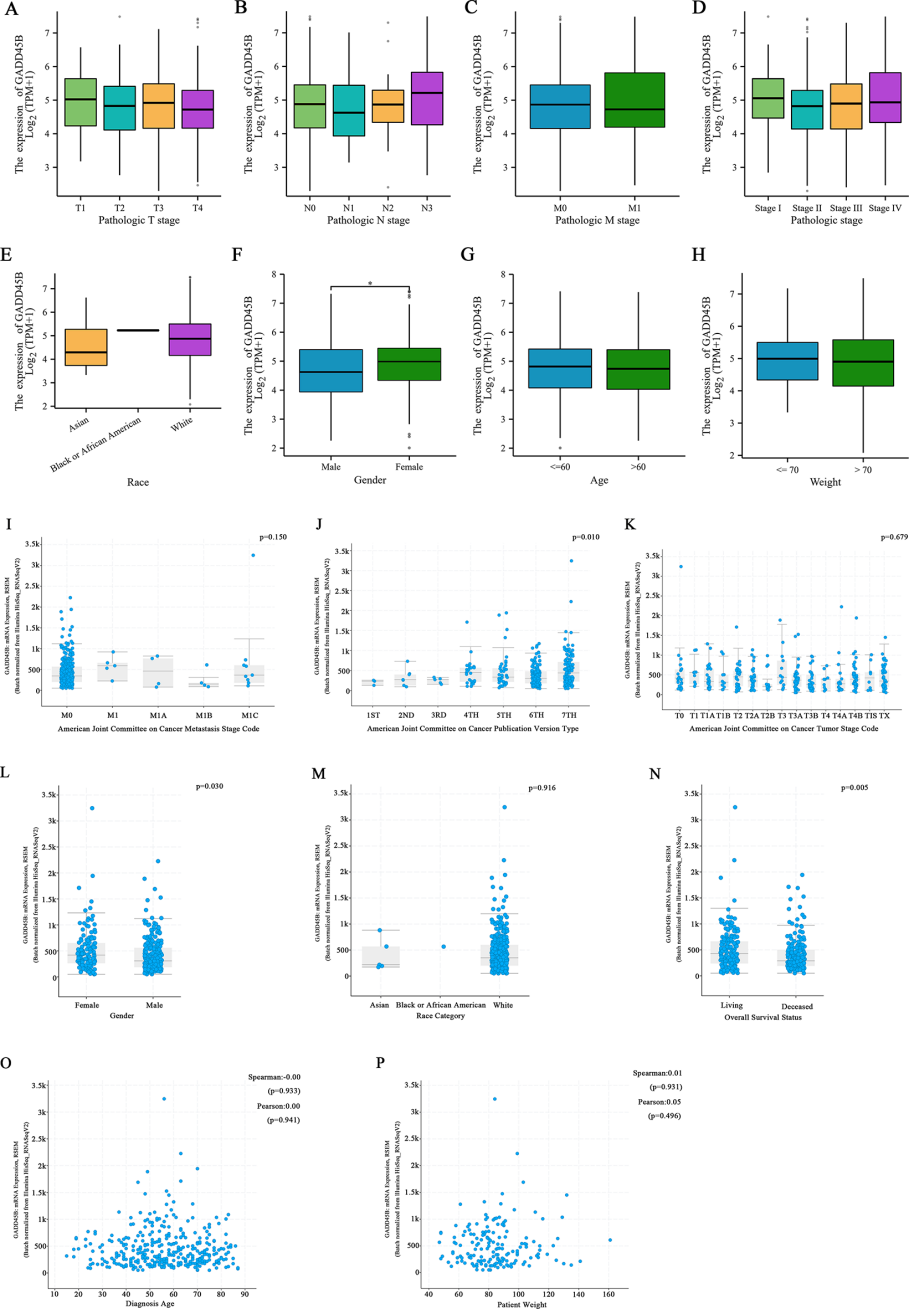
Expression of GADD45B in clinical factors in the TCGA-SKCM and cBioPortal databases. (**A-H**) GADD45B and SKCM clinicopathological features in the TCGA database. GADD45B did not differ by T, N, M, pathologic stage, race, age or weight but was higher in females. (**I-P**) GADD45B and SKCM clinical factors in the cBioPortal database. GADD45B did not differ in AJCC-metastasis and tumor stage, except AJCC-publication version stage. GADD45B also did not differ by patient race, age or weight but was higher in females and survivors (AJCC, American Joint Committee on Cancer)

Based on the data obtained from the TCGA and cBipPortal databases, we conducted an analysis to examine the relationship between GADD45B and various clinical factors in patients. Our findings indicate that there is no statistically significant difference in GADD45B expression based on the pathologic T, N, or M stage or the pathological stage (Fig. 2A-D). Furthermore, there were significant variations in GADD45B expression levels based on patient gender, but not based on race, age, or weight (Fig. 2E, G, H). Notably, GADD45B expression was found to be higher in females than in males (Fig. 2F). These results were consistent with the findings from the cBipPortal database (Fig. 2L, M, O, P). Similarly, GADD45B expression did not exhibit significant differences in relation to the AJCC-metastasis stage and AJCC-tumor stage, but it did show a positive correlation with the AJCC-publication version (Fig. 2I-K). Interestingly, our analysis also revealed that GADD45B expression was significantly higher in survivors than in deceased individuals, which aligns with the results presented in Fig. 1H (Fig. 2N).

### Differentially expressed genes and GO/KEGG analysis of GADD45B in SKCM

**Fig. 3:**
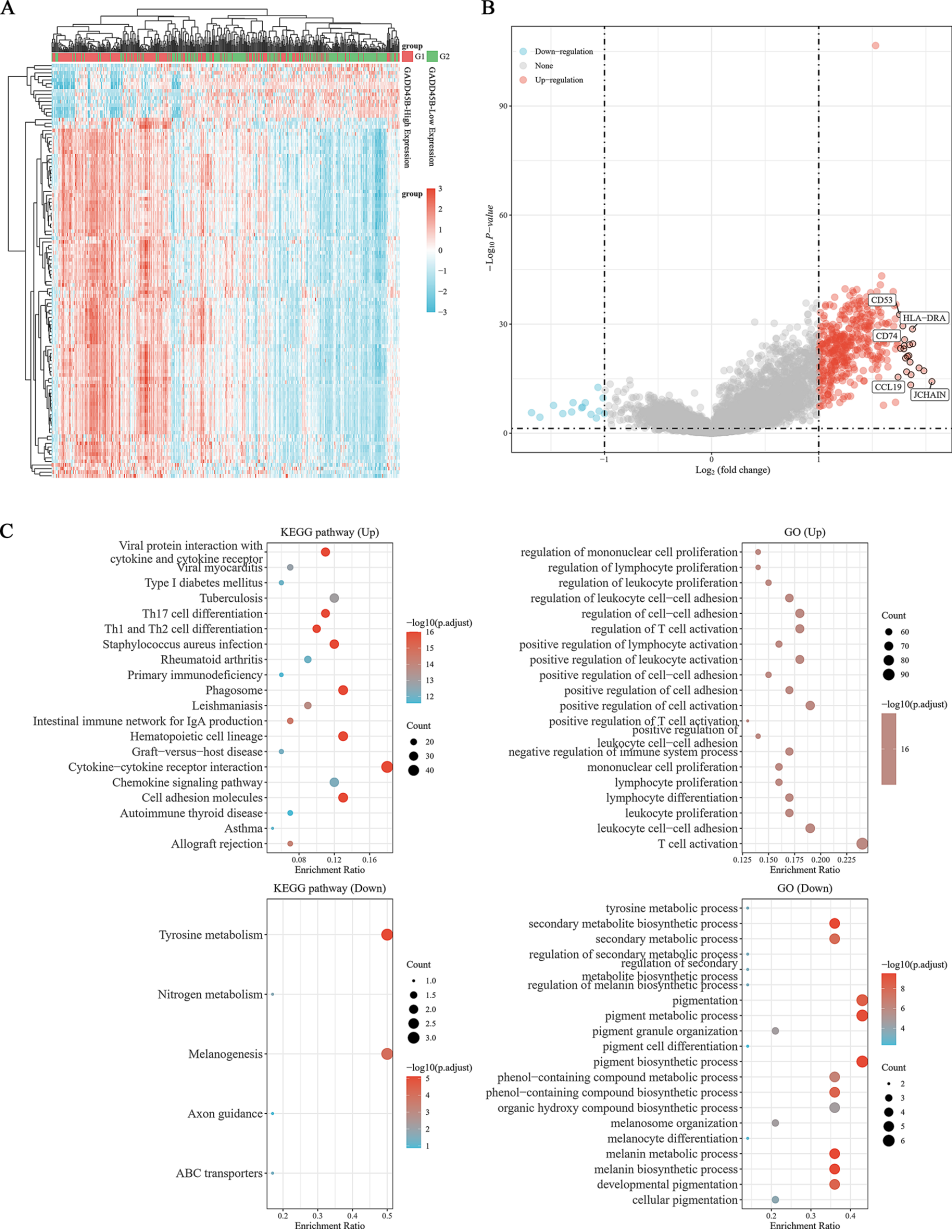
GO and KEGG analysis of GADD45B in SKCM. (**A**) Volcano plot of differential gene expression analysis (G1, GADD45B High Expression; G2, GADD45B Low Expression). (**B**) Heatmap shows differentially expressed genes. (**C**) KEGG and GO enrichment analyses. (|Log_2_FC|>1, adjusted *P* < 0.05)

The expression of GADD45B was examined using the LIMMA R package on the TCGA database to investigate its potential biological function in SKCM. This analysis revealed 423 differentially expressed genes (408 up, 15 down;|log2FC|>1, adjusted *p* < 0.05) (Fig. 3A, B). The GO and KEGG analyses indicated that the functions of GADD45B were associated with cytokine‒cytokine receptor interaction, tyrosine metabolism, T-cell activation, and pigment metabolic/biosynthetic process, among others (Fig. 3C). Based on these findings, in conjunction with previous reports, it can be speculated that GADD45B may have a close relationship with the occurrence and progression of SKCM, and it may also play a role in the immune response.

### GADD45B is positively correlated with cell inflammation and apoptosis but negatively associated with cell proliferation

**Fig. 4:**
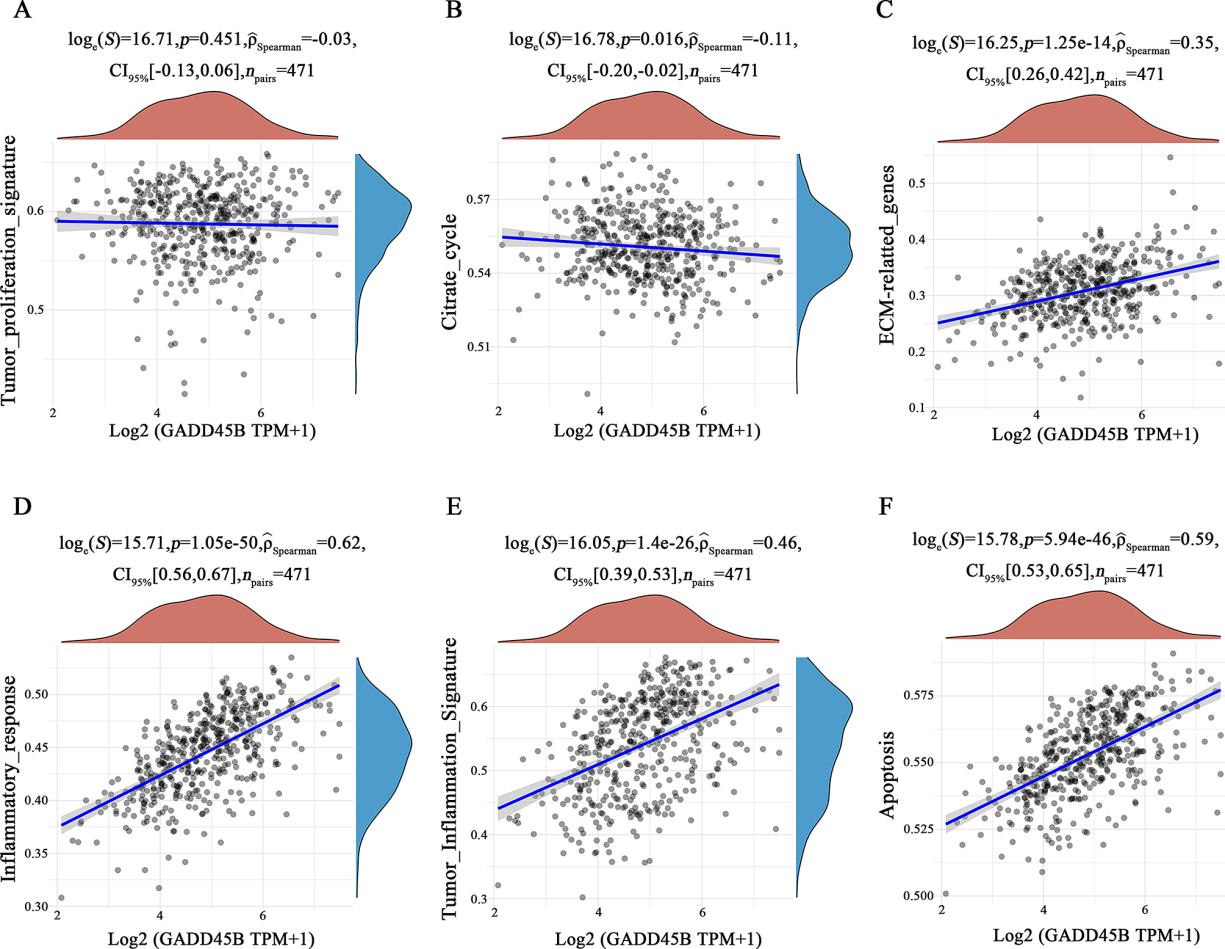
Evaluation of the correlation between GADD45B and tumor progression. (**A**, **B**) GADD45B was negatively correlated with tumor proliferation and the citrate cycle. (**C-F**) GADD45B was positively correlated with ECM-related genes, inflammatory response, tumor inflammation signature and apoptosis

Previous GO and KEGG analyses showed that GADD45B may be related to the development of SKCM, so we further analyzed the correlation between GADD45B and tumor-related processes. Correlation analysis showed that GADD45B expression was negatively correlated with the proliferation signature and citrate cycle in SKCM (Fig. 4A, B) but positively correlated with ECM, inflammation and apoptosis (Fig. 4C-F). Therefore, we hypothesized that GADD45B overexpression could inhibit the proliferation and promote the apoptosis of cutaneous melanoma cells; however, further experiments are needed to verify this effect.

### GADD45B inhibited SKCM proliferation, migration, and invasion and promoted apoptosis and S-phase arrest

**Fig. 5:**
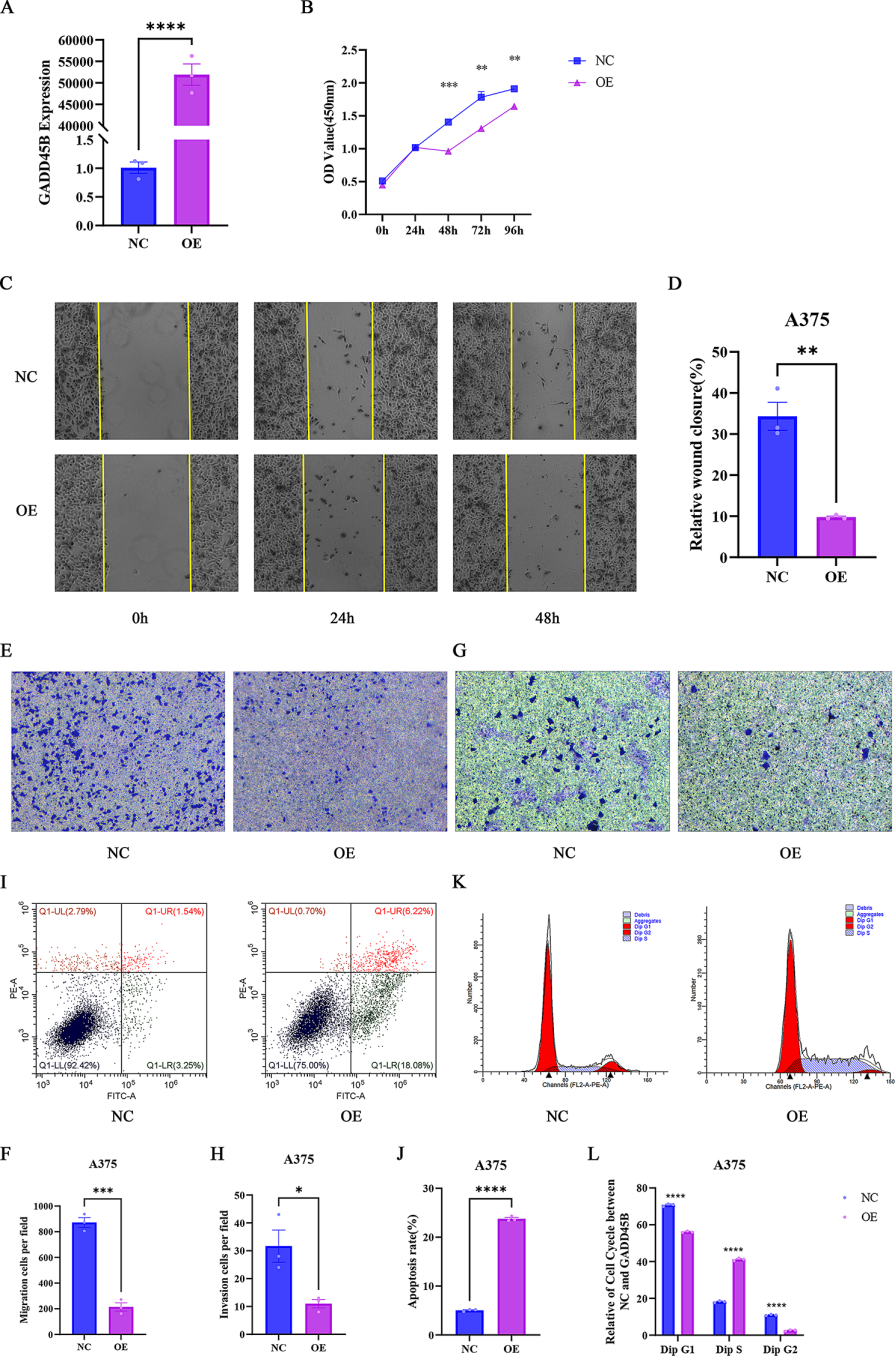
GADD45B inhibits proliferation, migration, and invasion and induces apoptosis and cell cycle arrest in SKCM cell lines. (**A**) RT‒qPCR detection of GADD45B overexpression in A375 cells. (**B**) GADD45B inhibits A375 cell proliferation according to the CCK8 assay. (**C-D**) GADD45B inhibits A375 cell migration, as determined by wound healing and (**E-F**) Transwell assays. (**G-H**) The invasion of A375 cells was assessed using Transwell assays. (**I-J**) Apoptosis and cell cycle (**K-L**) of A375 cells were examined through flow cytometry

To further investigate the impact of GADD45B on SKCM, we performed in vitro experiments using human malignant melanoma cell lines. Initially, GADD45B was overexpressed in both A375 and SK-MEL-1 cells, and the overexpression was confirmed through RT‒qPCR(Fig. 5A, Figure S1A). The CCK8 assay demonstrated that the overexpression of GADD45B significantly suppressed cell viability, with the most pronounced inhibition observed at 48 h (Fig. 5B). Furthermore, wound healing and transwell assays showed that GADD45B hindered cell migration (Fig. 5C-D) and was able to reduce cell invasion in a transwell invasion assay (Fig. 5E-H). These findings suggest that GADD45B may reduce viability and inhibit proliferation, migration, and invasion. As anticipated, the overexpression of GADD45B enhanced apoptosis (Fig. 5I, J, Figure S1B) and induced cellular S-phase arrest (Fig. 5K, L, Figure S1C) in SKCM cell lines.

**Fig. 6:**
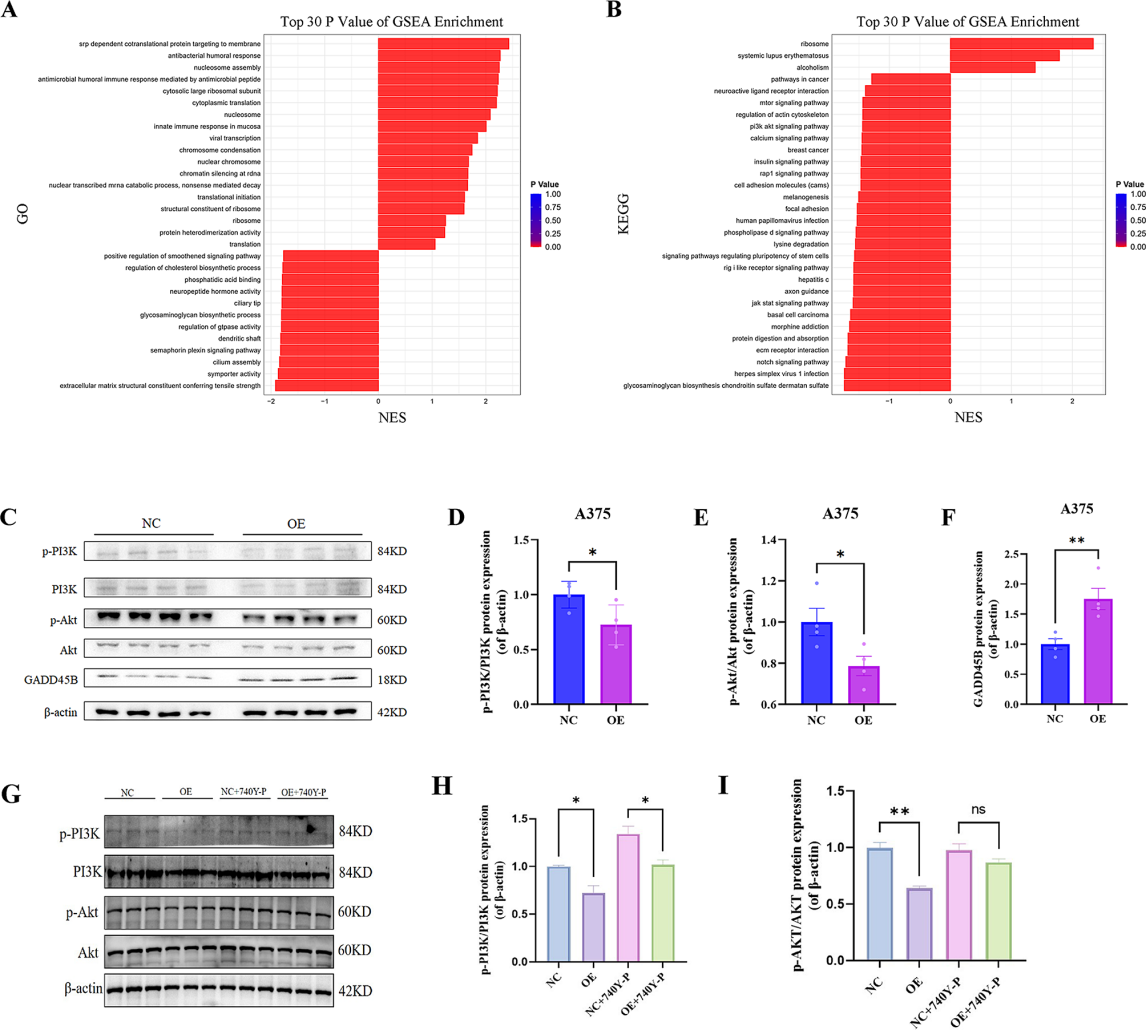
GADD45B inhibits PI3K-AKT signaling pathway activation. (**A-B**) GSEA-GO/KEGG enrichment bar graphs. (**C**) Quantification of p-PI3K/PI3K (**D**), p-Akt/Akt (**E**), and GADD45B (**F**) protein expression levels in A375 cells. The OE group showed a significant reduction in p-PI3K and p-Akt expression compared to NC (*p < 0.05, *p* < 0.01).(**G**) Western blot analysis of PI3K/Akt signaling pathway proteins after treatment with the PI3K inhibitor 740Y-P. (**H-I**) Quantification of p-PI3K/PI3K (**H**) and p-Akt/Akt (**I**) protein expression levels in NC, OE, NC + 740Y-P, and OE + 740Y-P groups. The 740Y-P treatment partially reversed the effects of GADD45B overexpression on PI3K and Akt phosphorylation (*p < 0.05, *p* < 0.01, ns = not significant)

In this study, we successfully achieved the overexpression of GADD45B in A375 cells, followed by the extraction of RNA for transcriptome sequencing. Through a comparison and enumeration of differentially expressed genes between the control group (N) and the overexpression group (G), our results revealed the presence of 160 upregulated genes and 540 downregulated genes (Figure S2A). Subsequently, we employed a clustering and analysis approach to construct a heatmap based on similarity of gene expression profiles among samples (Figure S2B). Furthermore, we utilized the GSE221101 sequencing data to perform GO/KEGG and GSEA analyses, which yielded results consistent with those obtained from the TCGA database (Figure S2C, D), and speculated that the PI3K/Akt signaling pathway might be associated with the regulation of A375 cell development by GADD45B (Fig. 6A). Western blot assays revealed that GADD45B was able to significantly lower the phosphorylation levels of PI3K and Akt proteins but not their total levels (Fig. 6B).

### In vivo validation of GADD45B function

**Fig. 7:**
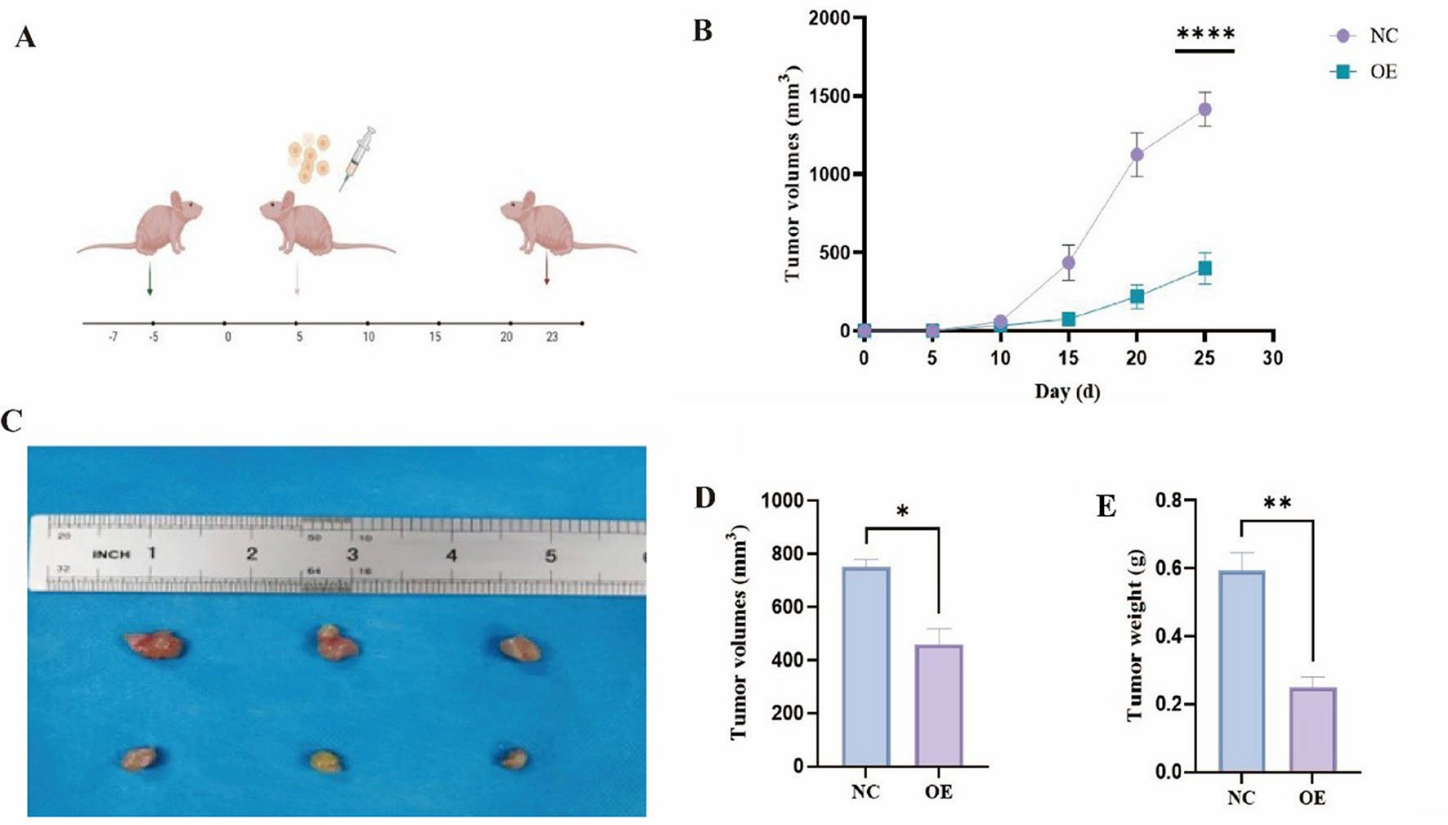
OE inhibits tumor growth in vivo. (**A**) Schematic of the in vivo xenograft model with tumor cell injection and monitoring timeline. (**B**) Tumor growth curves showing reduced tumor volume in the OE group compared to NC from day 15 (*p* < 0.0001). (**C**) Representative images of excised tumors, with smaller tumors in the OE group. (**D**) Tumor volume comparison at the endpoint. (**E**) Tumor weight comparison at the endpoint

To investigate the effect of OE on tumor growth in vivo, a xenograft mouse model was established. As shown in Fig. 7A, mice were subcutaneously inoculated with tumor cells on day 0, and tumor volumes were monitored every 5 days. The growth curves in Fig. 7B demonstrate that the tumor volume in the OE group was significantly smaller compared to the NC group from day 15 onwards. These results indicate that OE effectively suppresses tumor growth. Representative images of excised tumors from the NC and OE groups are presented in Fig. 7C, revealing visibly smaller tumor sizes in the OE group. Quantitative analysis showed that the average tumor volume in the OE group was markedly reduced compared to the NC group (Fig. 7D). Similarly, the average tumor weight was significantly lower in the OE group (Fig. 7E). These findings suggest that OE treatment effectively inhibits tumor growth in vivo, providing potential therapeutic value for cancer treatment.

### Association of GADD45B expression with macrophage-mediated immune responses

**Fig. 8:**
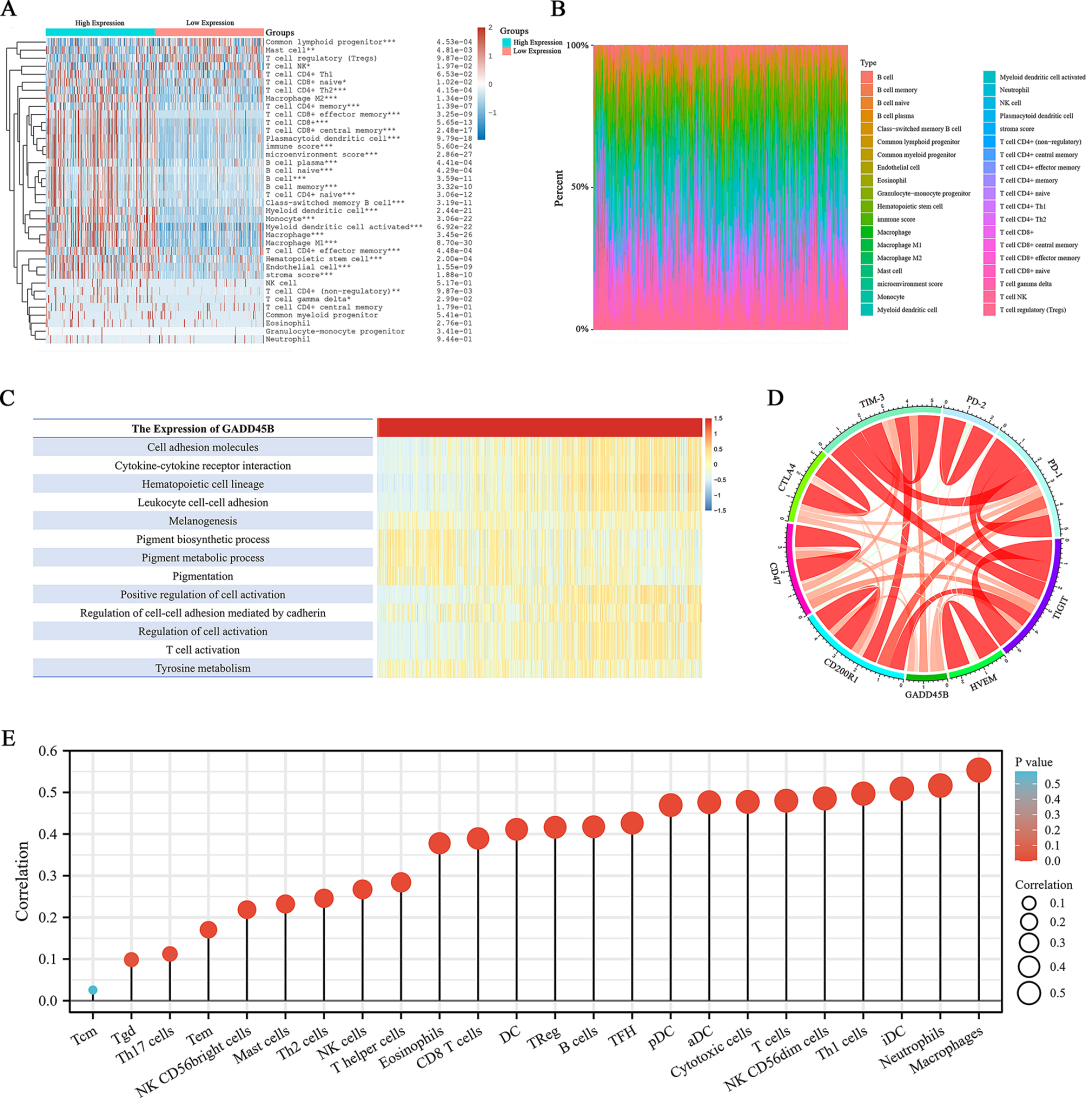
Correlation analysis of GADD45B and immune function. (**A**) Immunoscore heatmap of GADD45B in SKCM tissues and (**B**) percentage abundance of infiltrating immune cells. (**C**) Heatmap of GADD45B and immune function enrichment scores. (**D**) Pearson correlation of GADD45B with immune checkpoints. (**E**) Correlation analysis of GADD45B with individual immune cells

Due to the activation of lymphocytes and the release of chemokines and cytokines, cancer cells are killed, and the immune response is modulated, enhancing the antitumor immune effect [23, 24]. Hence, an investigation was conducted to examine the correlation between the expression of GADD45B and immune cells as well as related cytokines. RNA-sequencing data and clinical data for SKCM patients were obtained from TCGA. Immuneeconv was utilized to perform immune scoring and Spearman correlation analysis. The findings revealed the role of GADD45B in the SKCM immune response and interaction with B cells, macrophages, mast cells, T cells and others (Fig. 8A, B). GADD45B expression was positively correlated with cells such as macrophages, B cells, and myeloid dendritic cells (Figure S3A). Conversely, a negative correlation with cellular stem cells was analyzed by cell stemness (Figure S3B). The GSVA results showed that GADD45B expression was positively associated with the regulation of cell activation and the hematopoietic cell lineage but negatively correlated with pigment biosynthetic processes, among others (Fig. 8C). Additionally, GADD45B expression displayed a strong association with immune checkpoints (Fig. 8D). The immune score demonstrated that GADD45B expression was linked to various immune cells, with the highest enrichment observed in the association between GADD45B and macrophages (Fig. 8E). These findings suggest that GADD45B expression is connected to immune cell activity and immunotherapy responses in SKCM patients, particularly macrophages. Furthermore, GADD45B may serve as a novel immune checkpoint.

### GADD45B is highly enriched in macrophages and promotes M1 polarization

**Fig. 9:**
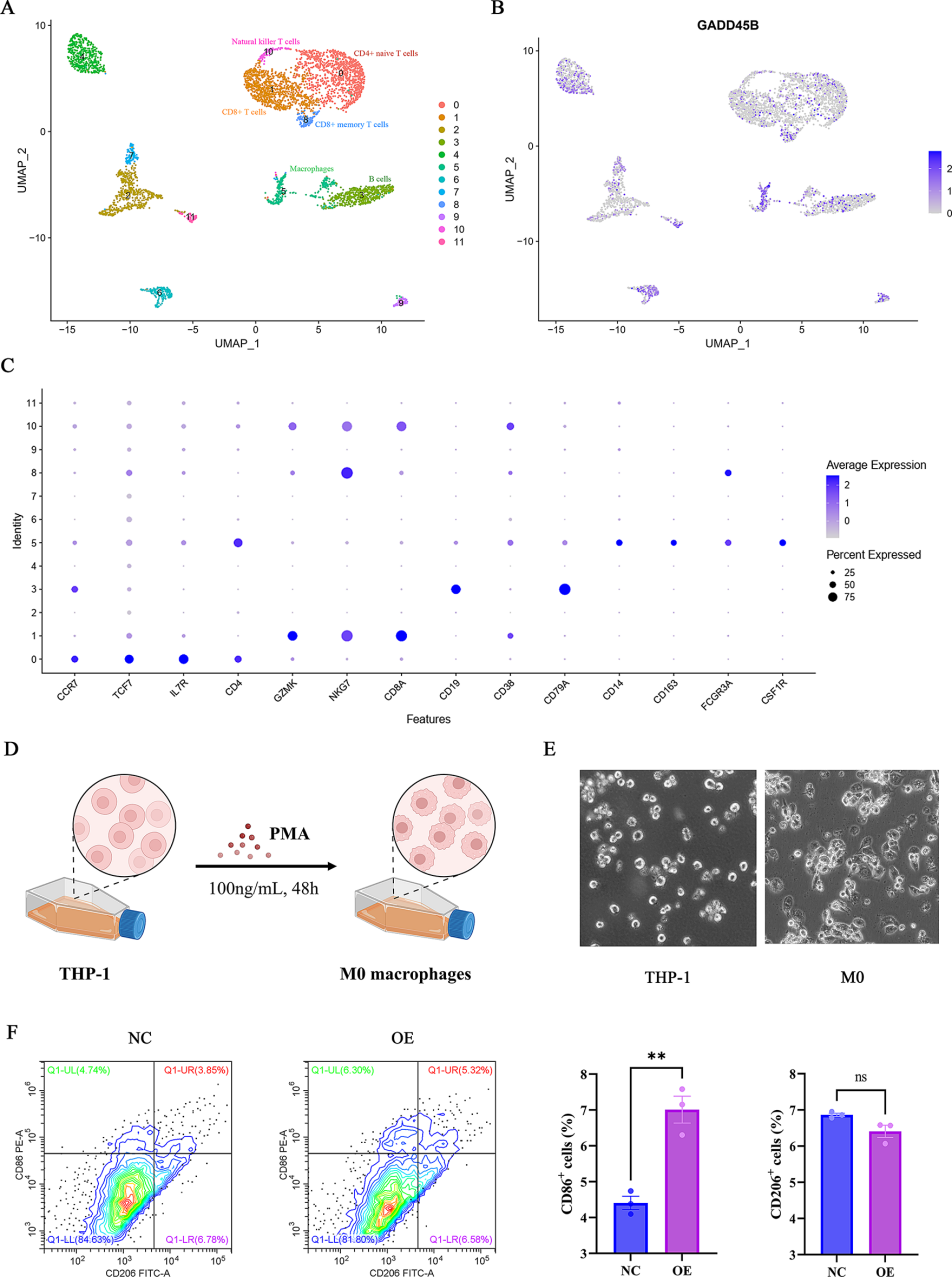
GADD45B expression pattern in SKCM lymphocytes and promotion of macrophage M1 polarization. (**A**) Subtypes in SKCM patients. (**B**) GADD45B is highly expressed in cluster 5. (**C**) Scatter plot of immune cell markers in each subtype. (**D**) Pattern of PMA-induced THP-1 cells as M0 macrophages. (**E**) Microscopy of THP-1 and M0 macrophages. (**F**) GADD45B promotes M1 polarization, as determined by flow cytometry

Single-cell sequencing is highly sensitive and accurate and is able to resolve cellular heterogeneity and reveal cellular subpopulation results. To carry out our analysis, we utilized the publicly available dataset from the GEO database and employed the R programming language to create 12 distinct clusters (Fig. 9A). Upon further examination, GADD45B was found to be highly enriched in cluster 5, showing robust expression signals (Fig. 9B). In addition, each cell cluster demonstrated distinct expression signatures, as illustrated by the expression distribution of cluster-specific marker genes (e.g., BCAN in cluster 2, APOC2 in cluster 4, SAA1 in cluster 6), providing a refined basis for cell-type annotation (Figure S4). Additionally, the presence of cellular markers such as CD14, CD163, FCGR3A, and CSF1R [19] within cluster 5 indicated the presence of macrophages (Fig. 9C, Figure S5). These findings align with the results presented in Fig. 7E.

Subsequently, we conducted cellular experiments to investigate the impact of GADD45B on macrophage polarization. M0 macrophages were derived by subjecting THP-1 cells to a 48 h treatment with 100 ng/mL PMA (Fig. 9D). This treatment induced a transition in cell morphology from the previously observed suspended ovoid shape to an adherent shuttle shape (Fig. 9E). To determine the differentiation status of the cells, we employed CD86 and CD206 markers, and flow cytometry analysis revealed that GADD45B exhibited the ability to enhance macrophage M1 polarization (Fig. 9F). Collectively, our findings suggest a potential association between GADD45B expression and immune regulation, particularly in macrophages.

### GADD45B expression is correlated with clinical drug sensitivity

**Fig. 10:**
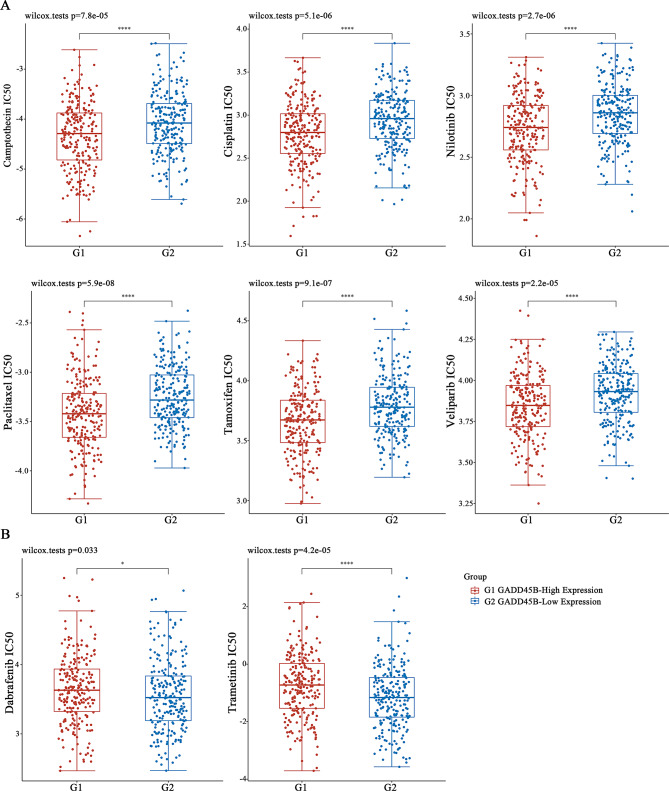
Evaluation of drug resistance between GADD45B and clinical agents. (**A**) GADD45B reduced the IC50 and resistance to clinical drugs, (**B**) but this was inconsistent across all drugs

Chemotherapeutic resistance frequently limits the efficacy of antitumor regimens received by cancer patients [25–27]. Consequently, the investigation of GADD45B expression in relation to drug resistance in SKCM patients was undertaken based on the GDSC database. Surprisingly, a negative correlation between GADD45B expression and chemotherapy resistance was observed in SKCM patients. The study observed elevated GADD45B expression associated with decreased IC50 for camptothecin along with six other chemotherapeutics (Fig. 10A), whereas IC50 rose correspondingly for dabrafenib and trametinib (Fig. 10B). Consequently, GADD45B expression could plausibly be related to augmented sensitivity of neoplastic cells toward clinically administered therapies, thereby contributing to extended cancer patient survival duration.

## Discussion

Melanoma is a highly malignant tumor originating from melanocytes, primarily occurring in the skin but also in the mucosal and visceral sites. Skin melanoma accounts for over 90% of diagnosed melanoma cases [28]. The incidence and mortality of SKCM have increased yearly, and the latest data show that cutaneous melanoma is the fifth most common cancer in terms of estimated new cases (6% in males, 5% in females) [29]. Environmental and genetic factors are involved in the pathogenesis of SKCMs, and ultraviolet (UV) radiation is the main environmental cause [30]. Aside from surgical resection, malignant melanoma lacks specific treatment, and patients have a poor prognosis [31]. Furthermore, tumor heterogeneity allows different patients to have different sensitivities to treatment, resulting in different clinical outcomes [32]. Accordingly, the exploration of additional biomarkers may substantially aid in the earlier identification and treatment of SKCM patients.

In this study, analysis of the TCGA-SKCM and CCLE databases revealed lower GADD45B expression in SKCM patients versus controls (*p* < 0.001), and clinical data corroborated these findings. Furthermore, we found that patients for whom the expression of GADD45B was higher had a more favorable prognosis (*p* < 0.05) and are expected to be diagnostic markers (AUC = 0.986). These findings reflect the good diagnostic and prognostic value of GADD45B for SKCM patients. On the basis of clarifying the clinical significance of GADD45B, its possible mechanism of action was further analyzed by bioinformatics.

GO/KEGG analysis showed that GADD45B was associated with immune cells and cell proliferation, differentiation, and adhesion. In addition, correlation analysis showed that GADD45B was positively correlated with inflammation and apoptosis but negatively correlated with tumor proliferation. Of course, further experiments are needed to verify this hypothesis.

Previous studies have shown that GADD45B plays an important role in the proliferation and migration of ovarian cancer [33], prostate cancer [34], esophageal cancer [35], non-small cell lung cancer [36] and other cancer cells. To investigate the effects of GADD45B on SKCM cells, A375 cells were used to successfully establish a cell line overexpressing GADD45B. CCK8 assays confirmed that GADD45B overexpression inhibited cell proliferation, migration and invasion, promoted apoptosis, and induced S-phase arrest. These findings were consistent with previous analytical results.

GADD4B is involved in tumor immunotherapy, and its deficiency can lead to autoimmune diseases and reduced efficiency of tumor immunodetection [10, 37]. Moreover, it has been reported that GADD45B can regulate the innate immune function of macrophages by regulating p38 and JNK signaling [19]. Macrophages play a crucial role in effectively inhibiting immunological checkpoints and the antitumor response [38–40]. GADD45B was linked to higher macrophage infiltration in this study, and it showed a positive regulatory association with the immune response, according to GO and KEGG analysis, GSVA, enrichment scoring, etc. Thus, GADD45B is expected to be a biomarker for novel immune examination. On the downside, because the data utilized in these studies were averages over numerous data samples, we were unable to gather information on the intrinsic heterogeneity of the tumor. This issue is effectively resolved by single-cell sequencing, which yields more accurate and sensitive data. Cell markers such as CD14, CD163, FCGR3A and CSF1R were used to identify macrophage clusters; CCR7, TCF7, IL7R, CD4, GZMK, NKG7 and CD8A were used to identify T-cell subpopulations; and CD19, CD38, and CD79A were used to identify B-cell clusters [41] (Fig. 8C, Figure S3). We also found that GADD45B was highly expressed in macrophage clusters specifically and distributed in both T and B-cell clusters. This is in line with the results in Fig. 7E. Therefore, we speculate that GADD45B may be involved in the immune response process during tumorigenesis and development, in which macrophages and other immune cells play a key role. The flow cytometry results indicated that GADD45B facilitated the polarization of macrophages from the M0 to M1 type, while no significant difference was observed for the M2 type. GADD45B is expected to be a novel immune check biomarker for SKCM.

At the same time, we cannot ignore that GADD45B is also distributed in other cell clusters (Fig. 8B) and further explored the expression of each cluster. The results showed that each gene was specifically expressed in each cell cluster, that is, BCAN (cluster 2) [42], TRIML2 (cluster 4) [43], TERT (cluster 6) [44], FAM3B (cluster 7) [45], CALCRC (cluster 9) [43], and COL1A1 (cluster 11) [43] (Figure S4). We were surprised to find that these genes have also been reported to serve as cellular markers for glial cells, germ cells, embryonic stem cells, cupula cells, gonadal endothelial cells, and mesenchymal precursor cells [42–45]. In addition, the association of GADD45B with neurology and embryos has also been reported.

X.Y. Shen [16] et al. reported that GADD45B is widely expressed in the nervous system and plays a vital role in epigenetics and neuroprotection. In addition, it mediates the proliferation of hippocampal neural stem cells after electroconvulsive shock induction, regulates neuronal differentiation, and may serve as a novel mediator of neuronal apoptosis in ischemic stroke [46–48]. X. Wu [49] et al. found that high expression of GADD45B improved the developmental efficiency of cloned pig embryos and played an essential role in the regulation of ovarian failure and embryonic development in mice [50]. Surely, this is not negligible in the epithelial-mesenchymal transition of renal tubular cells and in the regulation of DNA demethylation in embryonic stem cells [51, 52]. Interestingly, the above specifically expressed genes have also been reported in melanoma. For example, BCAN is significantly differentially expressed in IFN-treated melanoma, and promoter mutations of TERT in melanoma and COL1A1 promote melanoma invasion, among others [53–55]. In short, GADD45B may be associated with these specific genes, either in melanoma or in neural, embryonic, etc. Additionally, this provides ideas and references for future related studies.

It is now well recognized that tumor-associated macrophages influence tumor progression and metastasis and that the promotion of migration and invasion of cancer cells and resistance to anticancer treatments is usually associated with the M2 polarization of macrophages [56–58]. Tumor-derived exosomes (TDEs) have been shown by S.M. Morrissey [59] et al. to activate PD-L1, polarize macrophages to an immunosuppressive phenotype, and produce large quantities of lactic acid. These actions create an immunosuppressive milieu and facilitate tumor spread. In addition, studies have reported that the process of epithelial-mesenchymal transition (EMT) can generate tumor-initiating cells (TICs), which include cancer stem cells (CSCs) and cancer progenitors [60]; however, H.H. Lu [61] et al. found that EMT can promote the formation of mesenchymal properties of cancer cells and increase the ability of CSCs. Subsequently, we further explored the connection between GADD45B and CSCs. Stemness analysis showed a negative correlation between GADD45B and CSCs, and when GADD45B expression was upregulated, the mRNAsi score decreased significantly, which is also consistent with this theory. Therefore, inhibition of the proliferation and migration of SKCM by GADD45B may be associated with a reduction in cell stemness. Of course, this also needs to be further explored and verified by experiments.

The amalgamation of immune treatment and chemical therapy (chemotherapeutic immunization) has manifested superb healing impacts in clinical cancer patient care [62, 63], but adverse reactions such as drug resistance significantly limit the therapeutic efficacy of the combination strategy [64–66]. Furthermore, it has been shown that the establishment of an immunosuppressive niche lowers the curative effect of immunotherapy and increases the likelihood of drug resistance [67, 68]. Thus, we investigated GADD45B’s involvement in clinical medication resistance. The findings demonstrated that six clinical antitumor drugs, namely, camptothecin, cisplatin, nilotinib, paclitaxel, tamoxifen, and veliparib, had lower IC50 values when high GADD45B expression was present. As a result, high GADD45B expression improved drug sensitivity in cells and decreased the likelihood of clinical drug resistance. In contrast, we also observed that GADD45B overexpression did not reduce the IC50 values of dabrafenib and trametinib. Of course, this may be related, among other things, to the heterogeneity of clinical patients. Precision medicine is the implementation of tailored healthcare for patients who make clinical decisions based on their intrinsic biological information as well as clinical signs and symptoms (e.g., physiological, biochemical, behavioral, etc.); this is also known as personalized medicine [69, 70]. The relationship between GADD45B and the IC50 of each clinical medication found in this study also provides a basis for clinical precision medicine, which of course needs to be further investigated and explored in clinical treatment.

Our study systematically and comprehensively validated the potential of GADD45B as a diagnostic/prognostic marker for SKCM. Mechanistically, GADD45B may exert anticancer effects by regulating macrophage viability and participating in tumor immunotherapy. Even more fascinating to us was the finding that resistance to clinical oncology therapy was adversely connected with high expression of GADD45B; however, our study had several limitations. Since this work relies on publicly available data, although this study’s findings confirmed GADD45B’s role in tumor immune modulation and immunotherapy, more research is necessary to understand the underlying molecular process of GADD45B in SKCM.

Immunotherapy, particularly immune checkpoint inhibitors (ICIs) targeting PD-1, PD-L1, and CTLA-4, has revolutionized the treatment landscape for SKCM [7]. However, a substantial proportion of patients remain unresponsive or develop resistance to these therapies [65]. Our findings indicate that GADD45B is closely associated with immune cell infiltration, particularly macrophages, and may serve as an immune-related prognostic biomarker. Given its role in macrophage polarization towards the M1 phenotype, GADD45B could potentially modulate the tumor immune microenvironment and enhance anti-tumor immunity [17].

Recent studies have demonstrated that macrophage-driven immune responses are crucial for the efficacy of ICIs, with M1 macrophages exerting pro-inflammatory and tumor-suppressive effects [39]. Our single-cell RNA sequencing and immune correlation analysis revealed that GADD45B expression positively correlates with macrophage activation, particularly M1 polarization, suggesting that it may augment the efficacy of ICIs by fostering a more immunogenic tumor microenvironment. Furthermore, GADD45B expression was found to be linked to the regulation of immune checkpoints, further underscoring its potential as a target for combination therapies [40].

In addition to its immunomodulatory role, GADD45B demonstrated a negative correlation with chemotherapy resistance. Our drug sensitivity analysis revealed that high GADD45B expression was associated with increased sensitivity to multiple chemotherapeutic agents, suggesting that it could serve as a biomarker for stratifying patients who may benefit from combination strategies involving both chemotherapy and immunotherapy [25]. Given the increasing emphasis on precision medicine, integrating GADD45B expression levels into clinical decision-making could optimize therapeutic outcomes and minimize adverse effects [69].

Despite these promising findings, further preclinical and clinical validation is required to establish GADD45B as a reliable biomarker for immunotherapy responsiveness in SKCM. Future studies should focus on elucidating the mechanistic underpinnings of GADD45B-mediated immune modulation, conducting in vivo experiments, and exploring its utility in prospective clinical trials [61, 71]. If validated, GADD45B could represent a novel therapeutic target, paving the way for personalized treatment approaches in SKCM patients.

## Conclusion

In this study, we found that GADD45B holds promise as a diagnostic and prognostic biomarker for SKCM patients. Mechanistically, our data suggest that it may participate in tumor immunotherapy by modulating immune cell function and reducing drug resistance emergence. Taken together, these results indicate that GADD45B could work as a valuable molecular marker to help guide SKCM treatment, potentially facilitating the development of advanced immunotherapies with meaningful clinical impact going forward.

## Electronic supplementary material

Below is the link to the electronic supplementary material.

Supplementary Material 1

## Data Availability

No datasets were generated or analysed during the current study.
