# Supplementary material for: Integrated bioinformatic analysis identifies GADD45B as an immune-related prognostic biomarker in skin cutaneous melanoma
Source: Hereditas. 2025 May 11;162:74. doi: 10.1186/s41065-025-00437-0 (PMC12067689; doi:10.1186/s41065-025-00437-0)

**Figure S1. GADD45B promotes apoptosis and cell cycle arrest in SK-MEL-1 cells.** (A) The overexpression of GADD45B in SK-MEL-1 cells was confirmed by RT‒qPCR. (B, C) The apoptosis and cell cycle of SK-MEL-1 cells were detected by flow cytometry.


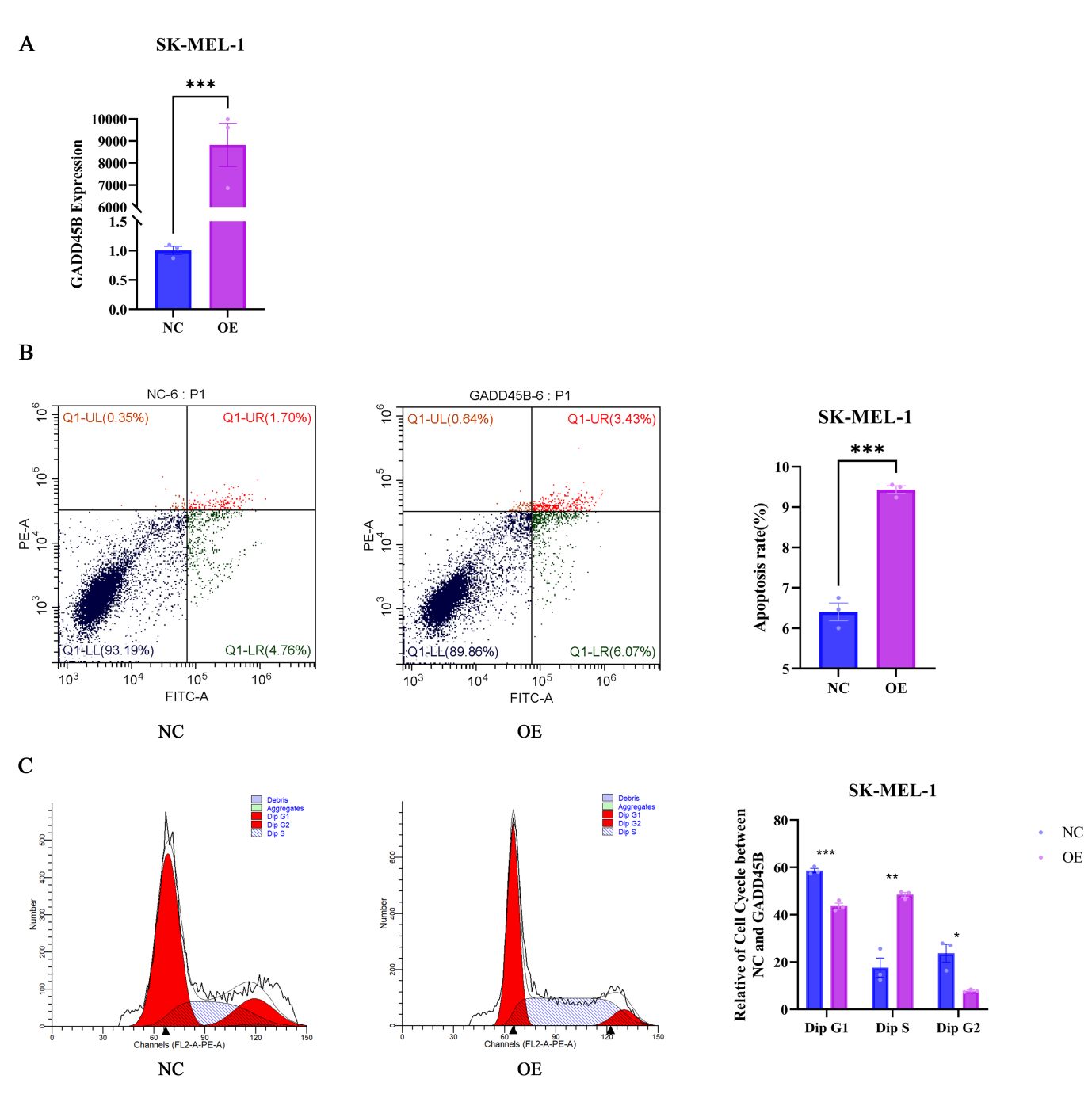


**Figure S2. Transcriptome sequencing analysis of GADD45B in the SKCM cell line A375.** (A) Volcano plots of differentially expressed genes (|Log2FC| >1, adj. P < 0.05). (B) Heatmap shows test vs. control samples horizontally and genes vertically (“G1, G2, G3”, OE-GADD45B, n = 3; “N1, N2, N3”, NC-GADD45B, n = 3). (C-D) GO/KEGG enrichment circle diagrams.


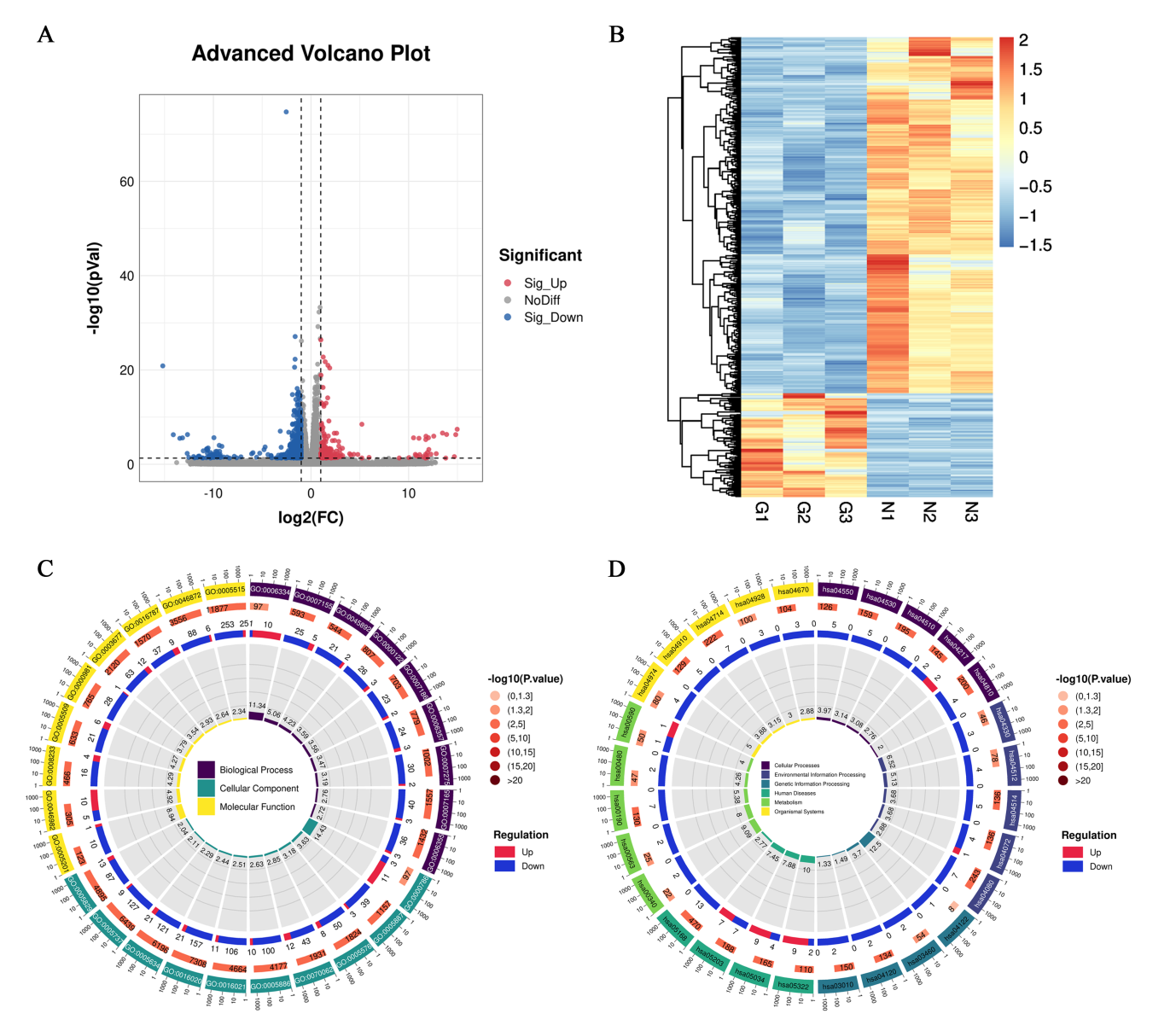


**Figure S3. Assessing the relationship between GADD45B and immune cells.** (A) The correlations between GADD45B expression and immune score were analyzed with Spearman’s test. (B) The distribution of mRNAsi scores in different groups.


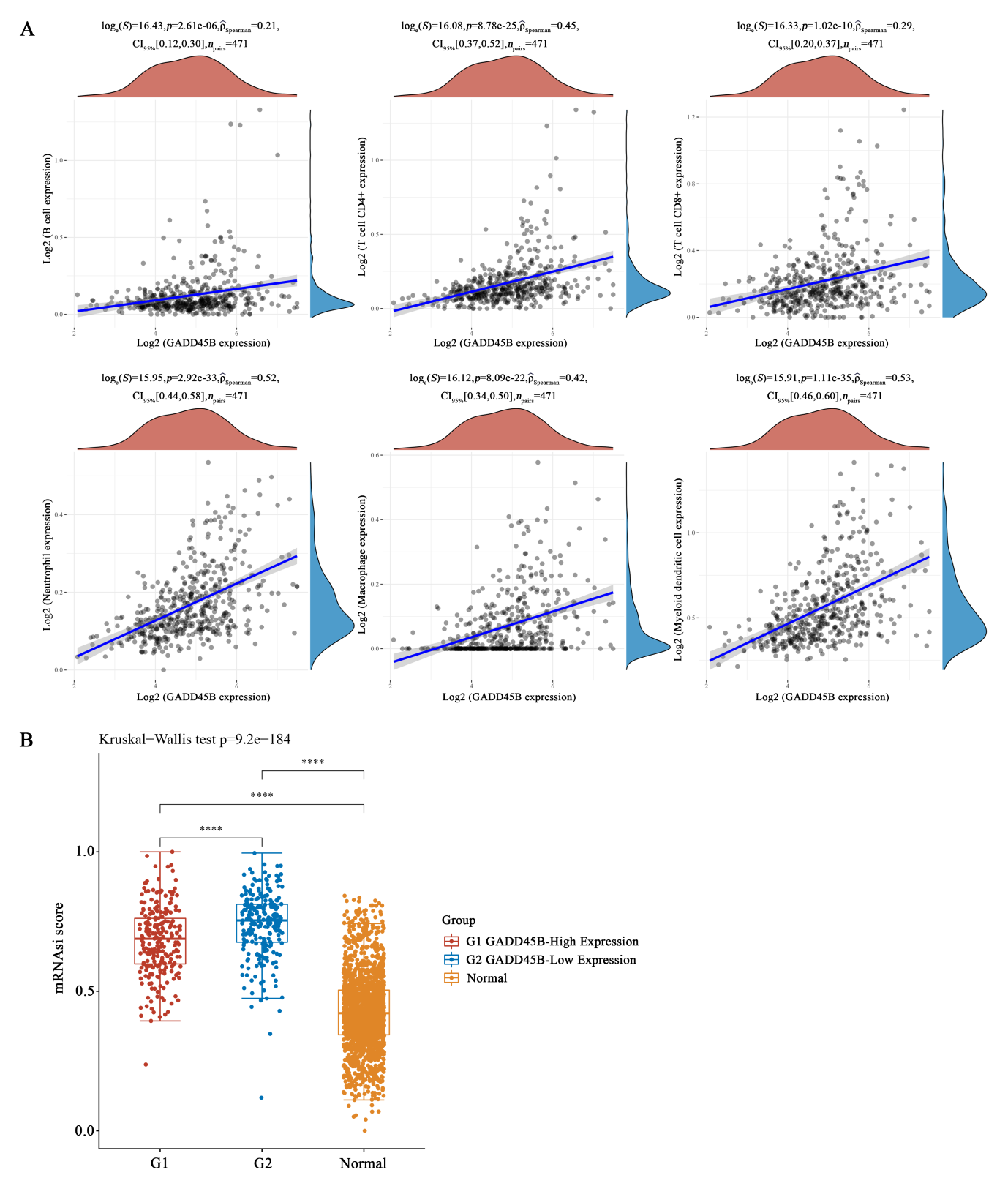


**Figure S4. Expression distribution and scatter plot of cellular markers analyzed by single-cell sequencing.** CD14, CD163, FCGR3A and CSF1R were used to identify macrophage clusters. CCR7, TCF7, IL7R, CD4, GZMK, NKG7 and CD8A to identify T-cell subpopulations. CD19, CD38, CD79A were used to identify B-cell clusters.


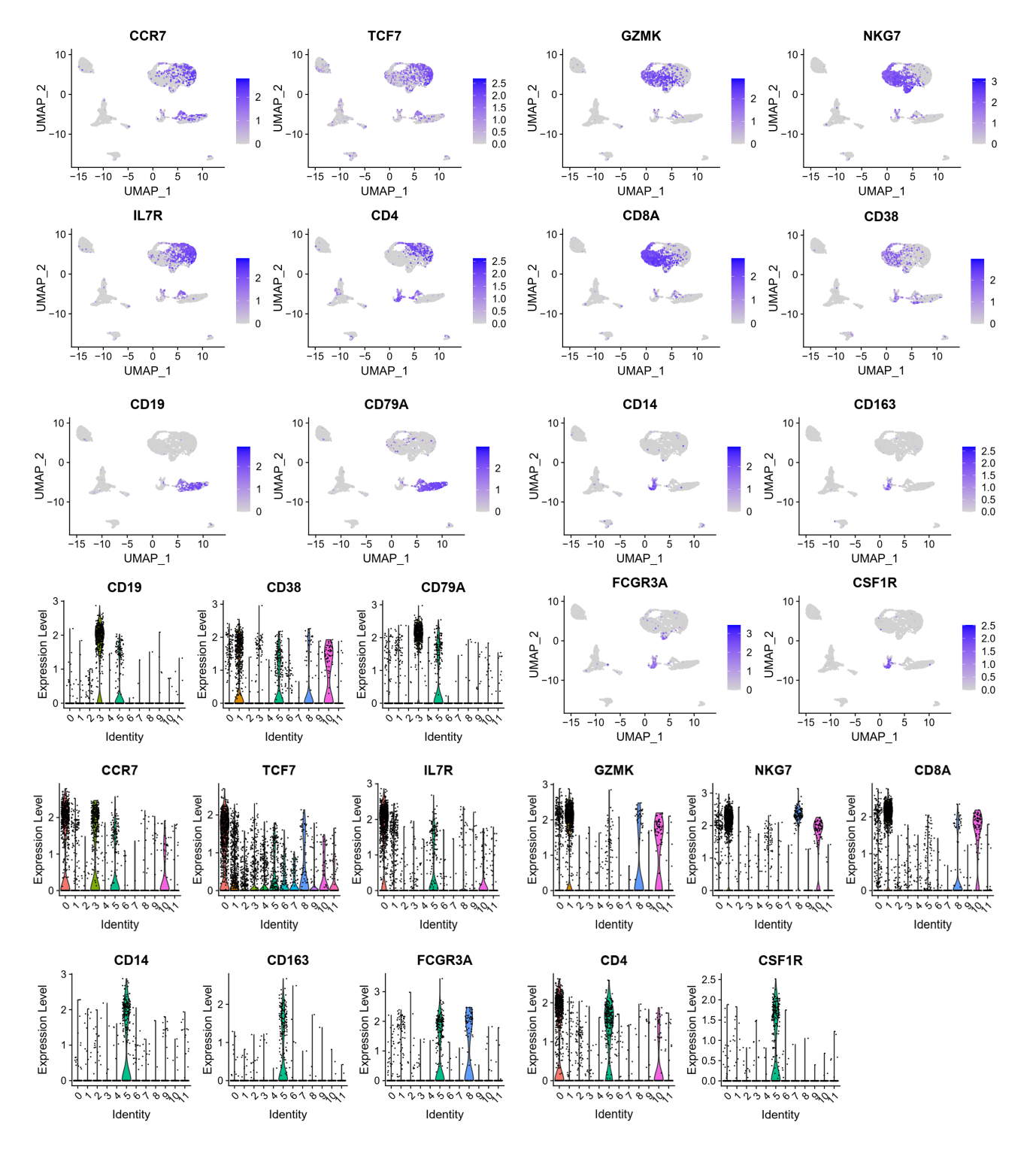


**Figure S5.** **Expression distribution and scatter plot of each cell cluster-specific highly expressed gene.** (A-C) Expression plots for significant enrichment of different genes in each cluster. BCAN, CA14 and HSPA2 (cluster 2); APOC2, TRIML2 and RDH5 (cluster 4); SAA1, AQP1 and TERT (cluster 6); GJC3, FAM3B and MT1L (cluster 7); CLDN5, SDPR and CALCRC (cluster 9); ISLR, COL1A1 and DCN (cluster 11).


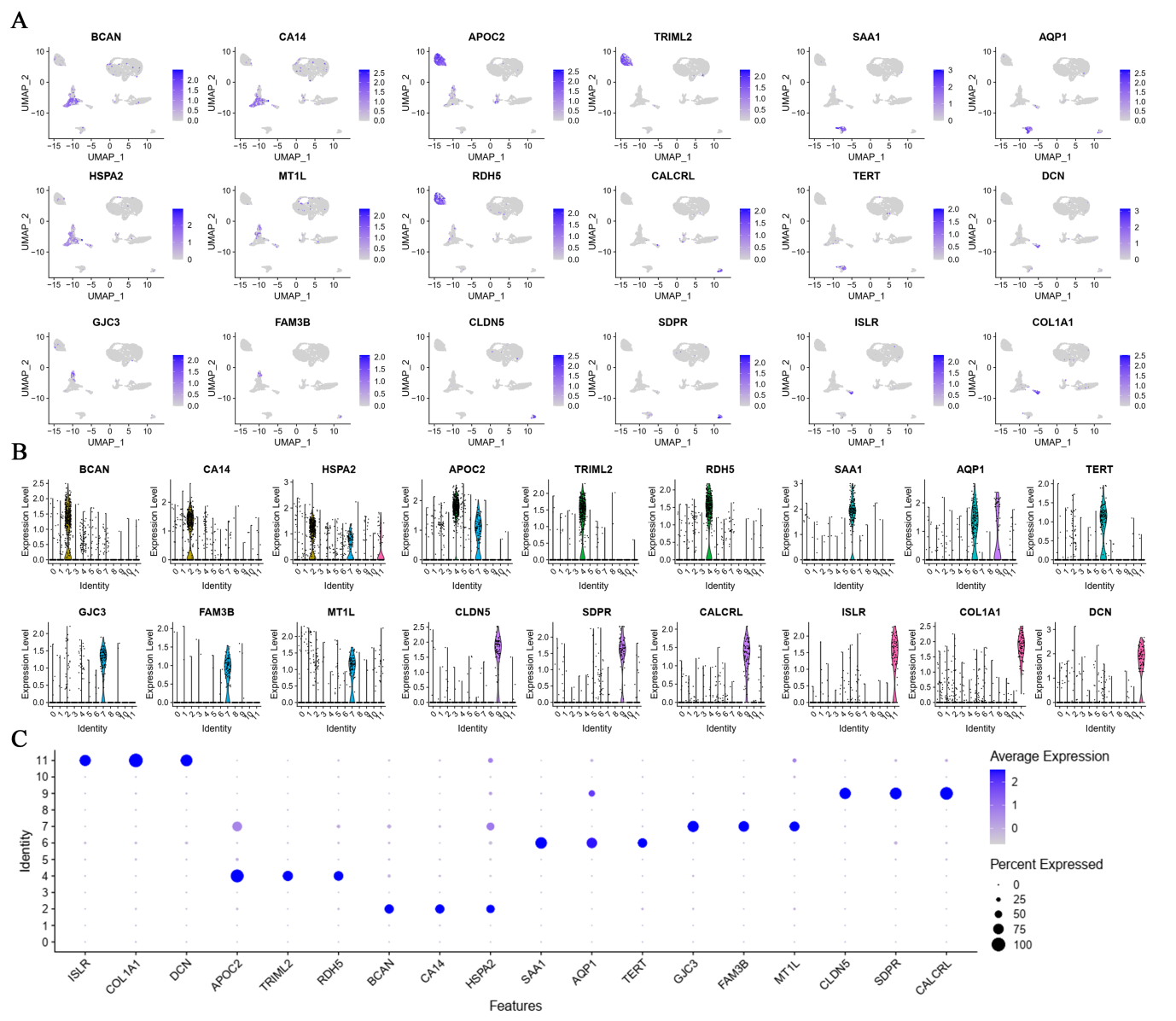

Supplement: Supplementary file 1 — Supplementary Material 1 [file 41065_2025_437_MOESM1_ESM.docx]
